# Metabolic Engineering of *Escherichia coli* for Ectoine Production With a Fermentation Strategy of Supplementing the Amino Donor

**DOI:** 10.3389/fbioe.2022.824859

**Published:** 2022-01-25

**Authors:** Hao Zhang, Zhong Liang, Ming Zhao, Yanqin Ma, Zhengshan Luo, Sha Li, Hong Xu

**Affiliations:** ^1^ State Key Laboratory of Materials-Oriented Chemical Engineering, Nanjing Tech University, Nanjing, China; ^2^ College of Food Science and Light Industry, Nanjing Tech University, Nanjing, China; ^3^ Jiangsu National Synergetic Innovation Center for Advanced Materials, Nanjing Tech University, Nanjing, China

**Keywords:** ectoine, *Escherichia coli*, metabolic engineering, medium optimization, amino donor

## Abstract

Ectoine, an osmotic pressure-compensated solute, is used in the food, agriculture, medicine, and cosmetics industries due to its ability to protect macromolecules. In this study, an ectoine-producing variant of *Escherichia coli*, ET08, was genetically constructed by introducing the *ectABC* gene cluster and eliminating metabolic pathways involving lysine and pyruvate. Medium optimization enhanced ectoine production from 1.87 to 10.2 g/L. Analysis of the transcriptional levels revealed that supplementation with ammonium sulfate enhanced the metabolic flux towards the biosynthesis of ectoine. Furthermore, by optimizing the copy number of *ectA*, *ectB*, and *ectC*, the recombinant *E. coli* ET11 (*ectA*:*ectB*:*ectC* = 1:2:1) produced 12.9 g/L ectoine in the shake flask and 53.2 g/L ectoine in a fed-batch fermenter, representing the highest ectoine titer produced by *E. coli*, which has great industrial prospects.

## Introduction

As a compatible solute, ectoine (4*S*-2-methyl-1,4,5,6-tetrahydro-4-pyrimidinecarboxylic acid) is commonly found in halophilic and halotolerant microorganisms and was first discovered in *Ectothiorhodospira halochloris* by Galinski et al. (1985). In addition to its primary function of maintaining cell osmotic balance and resisting the impact of high osmotic pressure, it also displays remarkable bioprotective properties, which have garnered increasing attention (Roychoudhury et al., 2012; Kunte et al., 2014; Hahn et al., 2017). There are many potential applications of ectoine in the food biotechnology, agriculture, skin care, and medical industries (Nakayama et al., 2000; Kentaro et al., 2005; Graf et al., 2008; Unfried et al., 2016; Boroujeni and Nayeri, 2018).

Ectoine has been synthesized from the precursor L-aspartate-β-semialdehyde (ASA) with L-2,4-diaminobutyrate transaminase (EctB), 2,4-diaminobutyrate acetyltransferase (EctA), and ectoine synthase (EctC) as catalysts (Göller et al., 1998; Calderon et al., 2004; Schwibbert et al., 2011; Li et al., 2017). These three enzymes are encoded by genes which are typically organized in the *ectABC* gene cluster, and may also comprise the *ectD* gene (Bursy et al., 2008). Similar gene clusters involved in ectoine biosynthesis were disclosed in *Marinococcus halophilus* (Louis and Galinski, 1997), *Halobacillus dabanensis* D-8T (Zhao et al., 2006), *Methylomicrobium alcaliphilum* 20Z (Reshetnikov et al., 2006), and *Nesterenkonia halobia* DSM20541 (Zhang et al., 2008).

Commercial ectoine production can be realized through the microbial fermentation of halophiles in a complex process called “bacterial milking” (Sauer and Galinski, 1998; Fallet et al., 2010). Although this method can be used to obtain ectoine on a large scale, the quantity of salt used corrodes the equipment. In addition, during the process, a higher resistance to osmotic pressure by the bacteria is required. To address the shortcomings of this process, great efforts have been made, including optimizing the process conditions and improving ectoine production performance by breeding halophilic bacteria. However, using transgenic non-halophilic bacteria for ectoine production has proven more efficient in recent years. For example, Becker et al. successfully integrated the *ectABCD* gene operon of *Pseudomonas stutzeri* A1501 into *Corynebacterium glutamicum* using systematic metabolic engineering, and then mutated aspartate kinase to ensure a sufficient supply of ASA. This resulted in a final overall spacetime yield of 6.7 g/L ectoine per day (Becker et al., 2013). In 2015, the *ectABC* gene cluster of *Halomonas elongata* DSM 2581 was overexpressed in *E. coli* K-12/BW25113. The overall production of ectoine was 25.1 g/L with aspartate and glycerol as substrates (He et al., 2015). Subsequently, an engineered strain of *E. coli*, ECT05, was constructed using a series of metabolic engineering strategies. The reported final titer was 25.1 g/L, and the overall yield of ectoine was 0.11 g/g of glucose (Ning et al., 2016). Recently, it was reported that ectoine production reached 65 g/L within 56 h, following transcriptional balancing of the ectoine pathway in *Corynebacterium glutamicum* (Gießelmann et al., 2019).

Although ectoine production can be realized by the fermentation of engineered microbial strains, low glucose conversion rate and low ectoine production efficiency are still observed. Therefore, recent reports have been primarily focused on the metabolic modification of engineered microbes. There are few studies on the nutritional optimization and fermentation regulation regarding engineered microbial strains. A study reported by Xu et al. revealed that through the regulation and optimization of the nitrogen source, the production of ε-poly-L-lysine was increased (Xu et al., 2018). The regulation of the nitrogen source was also reported to have a significant effect on ethanol and antibiotic production (Aharonowitz and Demain, 1979; Yue et al., 2012). This may also be applied to the synthesis of ectoine.

**FIGURE 1:**
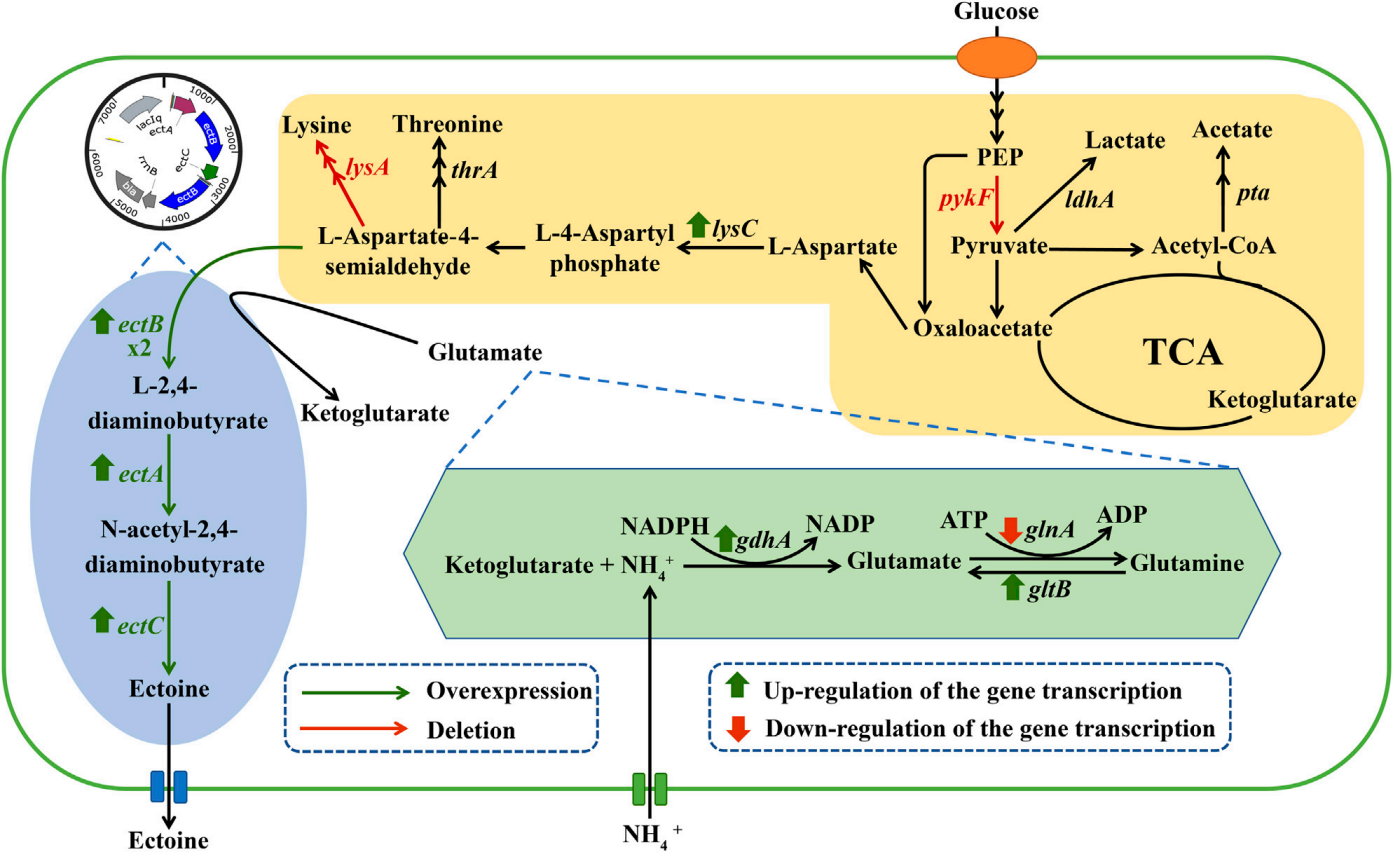
Related metabolic pathways for the synthesis of ectoine in engineered *E. coli*. The genes marked in green indicate the key genes of biosynthetic ectoine introduced into the engineered *E. coli*. The genes marked in red indicate deletion of the corresponding gene. Abbreviations: PEP, phosphoenolpyruvate.

In this study, we first constructed a variant of *E. coli* by introducing an ectoine biosynthetic pathway while eliminating the lysine synthesis branch and by-product metabolic pathways (Figure 1). Then, the effects of the nitrogen sources and amino donors on the production of ectoine were investigated. Next, the transcription levels of the key genes responsible for ectoine and ammonium metabolism were analyzed. Lastly, the copy number of *ectA*, *ectB*, and *ectC* were optimized to improve the final ectoine titer. This strategy provides a commercially attractive method for the microbial production of ectoine.

## Materials and Methods

### Strains, Plasmids, and Reagents

The strains and plasmids constructed and used in this study are listed in Table 1. All restriction digest enzymes, DNA polymerase and DNA ligase were obtained from Takara Bio Inc. (Dalian, China). Oligonucleotides were synthesized by GENEWIZ Bio Inc. (Suzhou, China). Ectoine was purchased from Sigma-Aldrich (Shanghai, China).

**TABLE 1:** Strains and plasmids used in this study.

Strains/plasmid	Relevant characteristic	Source
Strains		
*E. coli* MG1655	Wild type	This lab
*E. coli* BL21 (DE3)	Expression host	This lab
*Halomonas venusta* ZH	Wild type	This lab
ET00	*E. coli* BL21/pTrc-*ectABC*	This study
ET01	*E. coli* MG1655/pTrc-*ectABC*	This study
ET02	*E. coli* BL21/pET-*ectABC*	This study
ET03	*E. coli* MG1655/Δ*lysA*/pTrc-*ectABC*	This study
ET04	*E. coli* MG1655/Δ*thrA*/pTrc-*ectABC*	This study
ET05	*E. coli* MG1655/Δ*lysA*/Δ*thrA*/pTrc-*ectABC*	This study
ET06	*E. coli* MG1655/Δ*lysA*/Δ*pta*/pTrc-*ectABC*	This study
ET07	*E. coli* MG1655/Δ*lysA*/Δ*ldhA*/pTrc-*ectABC*	This study
ET08	*E. coli* MG1655/Δ*lysA*/Δ*pykF*/pTrc-*ectABC*	This study
ET08-N	*E. coli* MG1655/Δ*lysA*/Δ*pykF*	This study
ET09	*E. coli* MG1655/Δ*lysA*/Δ*pykF*/pTETA	This study
ET10	*E. coli* MG1655/Δ*lysA*/Δ*pykF*/pTETATA	This study
ET11	*E. coli* MG1655/Δ*lysA*/Δ*pykF*/pTETB	This study
ET12	*E. coli* MG1655/Δ*lysA*/Δ*pykF*/pTETBTB	This study
ET13	*E. coli* MG1655/Δ*lysA*/Δ*pykF*/pTETC	This study
ET14	*E. coli* MG1655/Δ*lysA*/Δ*pykF*/pTETCTC	This study
ET15	*E. coli* MG1655/Δ*lysA*/Δ*pykF*/pTETBTC	This study
Plasmid		
pTrc99a	*trc* promoter, cloning vector, Amp^r^	This lab
pET-28a	*T7* promoter, cloning vector, Kan^r^	This lab
pKD46	Temperature sensitive vector carrying red recombinase, Amp^r^	This lab
pKD3	Template vector, Cm^r^	This lab
pCP20	Temperature sensitive vector carrying FLP recombinase, Amp^r^	This lab
pTrc-*ectABC*	pTrc99a containing *Halomonas venuas ectABC* gene	This study
pET-*ectABC*	pET28a containing *Halomonas venuas ectABC* gene	This study
pTETA	pTrc99a, *trc*_*pro*_ *-ectABC-trc*_*pro*_-*ectA*	This study
pTETATA	pTrc99a, *trc*_*pro*_ *-ectABC-trc*_*pro*_-*ectA-trc*_*pro*_-*ectA*	This study
pTETB	pTrc99a, *trc*_*pro*_ *-ectABC-trc*_*pro*_-*ectB*	This study
pTETBTB	pTrc99a, *trc*_*pro*_ *-ectABC-trc*_*pro*_-*ectB-trc*_*pro*_-*ectB*	This study
pTETC	pTrc99a, *trc*_*pro*_ *-ectABC-trc*_*pro*_-*ectC*	This study
pTETCTC	pTrc99a, *trc*_*pro*_ *-ectABC-trc*_*pro*_-*ectC-trc*_*pro*_-*ectC*	This study
pTETBTC	pTrc99a, *trc*_*pro*_ *-ectABC-trc*_*pro*_-*ectB-trc*_*pro*_-*ectC*	This study

### Construction of Plasmids and Gene Knockout

The *ectABC* (MW414529) operon was amplified from the genome of *Halomonas venusta* ZH using the primers ET01-F and ET01-R. The PCR products were purified and then ligated into the linearized vector pTrc99a by in-fusion cloning with restriction sites of *Kpn* I and *Bam*H I, forming plasmid pTrc-*ectABC*. For the construction of the plasmid pET-*ectABC*, the *ectABC* was amplified by PCR with ET02-F and ET02-R using the genomic DNA of *H. venusta* ZH as a template. The PCR products were purified and then inserted into pET-28a by in-fusion cloning with restriction sites of *BamH* I and *Xho* I.

The genes (*ectA*, *ectB*, *ectC*) were amplified from the genome of *Halomonas venusta* ZH respectively. These gene fragments were fused with corresponding *trc* promoter using overlap-extension PCR to form *trc*_*pro*_-*ectA*, *trc*_*pro*_-*ectB*, and *trc*_*pro*_-*ectC*. The fragment *trc*_*pro*_-*ectA*, *trc*_*pro*_-*ectB*, and *trc*_*pro*_-*ectC* were assembled with linearized plasmid pTrc-*ectABC* (*Bam*H I/*Sal* I) using ClonExpress Entry One Step Cloning Kit to from plasmid pTETA, pTETB, and pTETC, respectively. A similar method can be used to construct a plasmid pTETATA, pTETBTB, pTETCTC, and pTETBTC.

Deletion of the *lysA*, *thrA*, *pykF*, *ldhA*, and *pta* genes were carried out using the Red recombination method (Datsenko and Wanner, 2000). The primers used for gene cloning and chromosomal manipulation are listed in Supplementary Table S1.

### Cultivation Conditions in Shake Flasks

The initial fermentation medium contained (per liter) 5 g glucose, 20 g yeast extract, 0.5 g NaCl, 2.5 g KH_2_PO_4_, 7.5 g K_2_HPO_4_, 1 g MgSO4·7 H_2_O. Seed cultures were carried out in 500 ml Erlenmeyer flasks containing 50 ml LB medium (10 g/L tryptone, 5 g/L yeast extract, and 10 g/L NaCl) at 37°C and 200 rpm for 8 h. The seed culture (5 ml; 10% v/v) was inoculated into a 500 ml baffled shake flask containing 50 ml initial fermentation medium and cultivated at 37°C and 200 rpm for 48 h. Both media were supplemented with 100 mg/L ampicillin. As the inducer, 0.1 mmol/L IPTG was added at 4 h. 20 g/L of glucose was supplied by adding feeding solution (500 g/L glucose) every 12 h during the fermentation process.

To optimize the types of organic nitrogen sources, five organic nitrogen sources (yeast extract, peptone, beef extract, soybean meal extract, corn steep liquor) were chosen for *E. coli* ET08 fermentation of ectoine. The concentrations of each nitrogen source were 20 g/L. Then, complex nitrogen sources were optimized by mixing various concentrations of yeast extract (10 g/L, 15 g/L, 20 g/L) up with ammonium chloride (152 mM) and sodium nitrate (152 mM) respectively. The medium without inorganic nitrogen addition was used as control check. To optimize the exogenous amino donor, the effects of various concentrations of ammonium chloride (0, 38, 76, 152, 228 mM), ammonium sulfate (0, 19, 38, 76, 114 mM), and sodium glutamate (0, 38, 76, 152, 228 mM) were investigated in shake-flask tests.

### Cultivation Conditions in a 7.5 L Bioreactor

Fed-batch fermentation of *E. coli* ET11 was carried out in a 7.5 L bioreactor (New Brunswick BioFlo/CelliGen 120, New Brunswick, Germany) containing 3.5 L medium. The fed-batch fermentation medium contained (per liter) 15 g glucose, 20 g yeast extract, 0.5 g NaCl, 2.5 g KH_2_PO_4_, 7.5 g K_2_HPO_4_, 1 g MgSO4·7 H_2_O, 10 g ammonium sulfate. The primary seed culture was prepared by transferring colonies from LB agar plate into a 50 ml shake flask containing 10 ml LB medium and culturing at 37°C and 200 rpm for 10 h. Then, the second seed culture was obtained by transferring 2 ml primary seed culture into a 1 L baled shake flask containing 200 ml LB medium and culturing under the same conditions. When the optical density at 600 nm (OD_600_) reached about 5, the second seed culture was inoculated into fermentation medium using a 10% (v/v) inoculum size. The pH was automatically controlled at 7.0 by adding NH_4_OH (25%, v/v), and the temperature was maintained at 37°C. The dissolved oxygen (DO) was maintained at 40% during 0–24 h by variation of the stirrer speed and the aeration rate. Subsequently, the DO was maintained at 20% from 24 to 48 h. The fed-batch culture was implemented in the early stage of fermentation (0–18 h), while the glucose concentration was maintained below 1 g/L by adding feeding solution (600 g/L glucose) at a designed rate in the middle-to-late period (24 h-end). As the inducer, 0.1 mmol/L IPTG was added when OD_600_ reached 0.4.

### RNA Sample Preparation and RT-qPCR Analysis

As the fermentation in shake flasks reached 36 h, the production was closed to maximum production. At this timepoint, the transcriptional levels of the key genes in ammonium metabolic pathways and ectoine synthesis were determined. Total RNA was isolated using RNAiso Plus (9108Q, TaKaRa Biotechnology Company, China). The synthesis of cDNA was then performed with PrimeScriptTM II 1st Strand cDNA Synthesis Kit (6210A, TaKaRa Biotechnology Company, China), using the total RNA as template. Real-time PCR (StepOnePlusTM Real-Time PCR System, Applied Biosystems, United States) was carried out with SYBR^®^ Premix Ex TaqTM (RR420Q, TaKaRa Biotechnology Company, China) as fluorochrome, with 16S rDNA as an endogenous control gene. The primers used for qPCR were designed according to the *E. coli* genome sequence and were summarized in Supplementary Table S2. The amplification program consisted of one cycle at 95°C for 30 s, followed by 40 cycles at 95°C for 5 s, 60°C for 30 s. All reactions were repeated three folds. Data from RT-qPCR were treated with the 2^−ΔΔCt^ method for relative quantification (Livak and Schmittgen, 2000). To present the results in a better way, the formula 2^−ΔΔCt^ was multiplied by 1. Therefore, the comparative expression level of each gene under the control was always 1 (Liu et al., 2017).

### Analytic Methods

Cell growth was monitored by measuring the absorbance at 600 nm (OD_600_). Dry cell weight (DCW) was calculated based on the calibration curve (OD600 = 2.6131DCW–6.4197). The concentration of extracellular ectoine was determined *via* high-performance liquid chromatography (HPLC) using TSKgel ODS-80Ts column (4.6 × 250 mm, Tosoh, Tokyo) with an acetonitrile/water mixture (2:98 v/v) at a flow rate of 0.5 ml/min as the mobile phase. Ectoine was monitored by a UV detector at a wavelength of 210 nm. The residual sugar in the fermentation broth was measured using an SBA-40C biological analyzer (Shandong Academy of Sciences, China). Organic acid was determined by HPLC, using an Aminex HPX-87H column (Bio-Rad, Hercules, United States) kept at 65°C, with 5 mM sulfuric acid as the mobile phase at a low rate of 0.5 ml/min (Dong et al., 2021).

## Results and Discussion

### Metabolic Construction of *E. coli* for Ectoine Production

**FIGURE 2:**
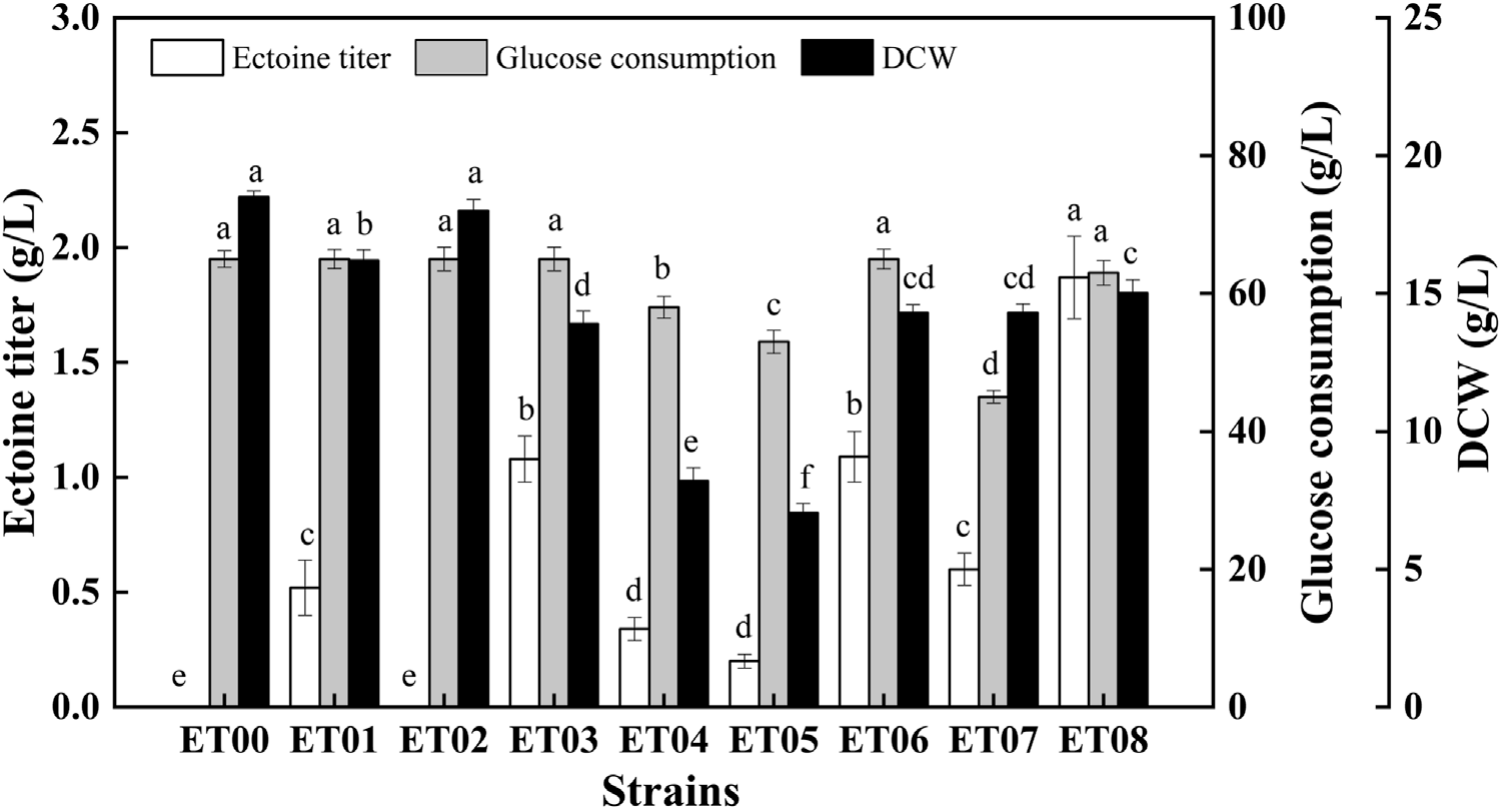
Comparison of ectoine titer, glucose consumption, and DCW in recombinant *E. coli* strains. Values represent the mean ± SD. Statistical analysis was performed by Duncan’s test (*p* < 0.05). Different lowercase letters indicate significant differences.

To achieve the heterologous synthesis of ectoine, two different host cells, *E. coli* MG1655 and *E. coli* BL21 (DE3), were chosen based on their genetic background and the maturity of the genetic tools. The constructed plasmid pTrc-*ectABC* was transferred into *E. coli* BL21 (DE3) and *E. coli* MG1655, respectively to construct the recombinant strains ET00 and ET01 (Table 1). The extracellular ectoine titer reached 0.52 g/L after 48 h cultivation using ET01 (Figure 2). However, the production of ectoine by ET00 was not detected (Figure 2) despite similar expression levels of *ectABC* in ET00 and ET01 (Supplementary Figure S1). To further improve the expression level of *ectABC*, the plasmid pET-*ectABC* was transferred into *E. coli* BL21 (DE3), to generate strains ET02. As expected, the expression of *ectABC* increased significantly, but ectoine was still not detected. Accordingly, *E. coli* MG1655 was a better chassis for the production of ectoine. The recombinant strain ET01 was chosen for further genetic manipulation.

### Elimination of Branch and By-Product Metabolic Pathways

To reduce L-aspartate-β-semialdehyde shunting via branch metabolism, the genes *lysA* (encoding diaminopimelate decarboxylase) and *thrA* (encoding aspartate kinase/homoserine dehydrogenase) in *E. coli* ET01 were knocked out individually and collectively, generating the recombinant strains *E. coli* ET03, *E. coli* ET04, and *E. coli* ET05, respectively. As shown in Figure 2, compared to *E. coli* ET01, the ectoine titer of *E. coli* ET03 (1.08 g/L) was increased 2.08-fold, however the biomass (14 g/L) decreased with the same glucose consumption (65 g/L). When *thrA* was deleted, the ectoine titer of *E. coli* ET04 was decreased to 0.34 g/L, while glucose consumption and cell growth showed a similar trend, declining to 58 g/L and 8.2 g/L, respectively. Similarly, as *lysA* and *thrA* were knocked out collectively, the ectoine titer of *E. coli* ET05 decreased (0.22 g/L) with 53 g/L of glucose consumption and 7.1 g/L of DCW. The results indicated that the deletion of *thrA* significantly inhibited the growth of *E. coli* ET01, which was consistent with previous reports (Ning et al., 2016). The deletion of *thrA* might increase the metabolic burden of cells (Fang et al., 2014), which further affected the growth of the strain and synthesis of ectoine.

In addition to ectoine, acetate, as a main organic acid, was observed in the broth (Dong et al., 2021). Metabolic pathway analysis showed that acetate and lactate are derived from pyruvate (Figure 1). Since the production of organic acids distributes the carbon flux and can significantly inhibit cell growth by reducing the pH level, this may have affected the final titer. As such, to further increase the final quantity of ectoine, recombinant strains *E. coli* ET06, *E. coli* ET07, and *E. coli* ET08, were constructed by deleting genes of *pta*, *ldhA*, and *pykF* in *E. coli* ET03, respectively. which are responsible for acetate, lactate, and pyruvate synthesis, respectively (Figure 1). When *pta* was deleted, the ectoine production and biomass of *E. coli* ET06 were similar to those of *E. coli* ET03 but with an extended lag phase. Although the deletion of *pta* significantly reduced the titer of acetate from 6.52 g/L to 1.81 g/L in the broth, the titers of lactate, and pyruvate increased significantly (Supplementary Figure S2). Meanwhile, the ectoine titer of *E. coli* ET07 was decreased to 0.61 g/L with the lowest glucose consumption (45 g/L). These results indicated that the deletion of *pta* and *ldhA* had no positive impact on ectoine production. However, the ectoine titer of *E. coli* ET08 was increased by 73.1% (reached to 1.87 g/L) compared with that of *E. coli* ET03. A slight increase in the biomass (15 g/L) was observed in *E. coli* ET08, which indicated that the deletion of *pykF* can promote the growth of the strain. Organic acid analysis showed that acetate (3.25 g/L) was reduced by 50.2%, while lactate and pyruvate no longer accumulated (Supplementary Figure S2). This might explain the reason for the increase in biomass. In addition, the *pykF* deficient decreased the flux from phosphoenolpyruvate (PEP) to pyruvate and increased the PEP pool (Siddiquee et al., 2004). The accumulation of PEP, as a prerequisite for ectoine synthesis, might further promote the synthesis of ectoine. Based on these results, *E. coli* ET08 was chosen as the potential strain for further studies.

### Optimization of Nutritional Element to Improve the Ectoine Production

**FIGURE 3:**
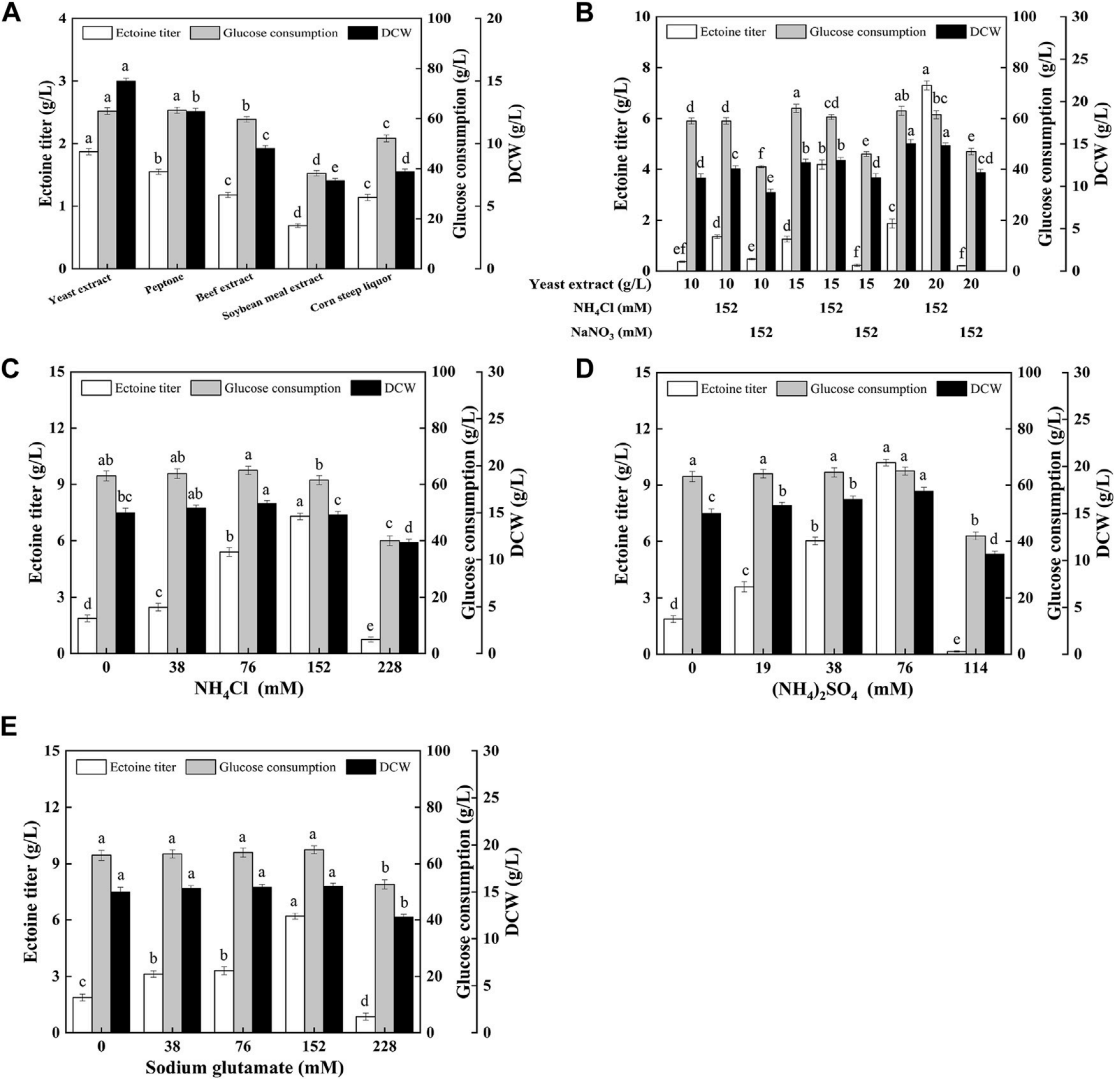
Effects of **(A)** different organic nitrogen sources, **(B)** the yeast extract mixed with inorganic nitrogen, **(C)** different ammonium chloride concentrations, **(D)** different ammonium sulfate concentrations, **(E)** different sodium glutamate concentrations on ectoine production, glucose consumption, and cell growth in *E. coli* ET08. Values represent the mean ± SD. Statistical analysis was performed by Duncan’s test (*p* < 0.05). Different lowercase letters indicate significant differences.

To further improve ectoine production, it is necessary to optimize the type and quantity of the nitrogen sources. As shown in Figure 3A, the yeast extract was more conducive to ectoine synthesis and bacterial growth than other organic nitrogen sources. In addition, as shown in Figure 3B, with the increase in the concentration of the yeast extract concentration in the medium, the production of ectoine by *E. coli* ET08 also increased, except for the titer obtained by sodium nitrate addition. The production of ectoine (7.29 g/L) was achieved with the supplementation of 20 g/L yeast extract and 152 mM ammonium chloride, which was 3.90-fold higher than that of the control (1.87 g/L). With the same yeast extract concentration, the ectoine titer with the addition of extra ammonium chloride was significantly higher than that of the blank control and sodium nitrate. The combination of the yeast extract and nitrate did not have a positive effect on the production of ectoine. Both the biomass and sugar consumption of *E. coli* ET08 were also decreased (Figure 3B). This result implied that ammonium salt played a more significant role in promoting the production of ectoine compared to nitrate salt. In addition, the ammonium salt was more conducive to microbial absorption than the nitrate, and may act as an exogenous amino donor by providing the NH_4_^+^ ions involved in the synthesis of glutamate (Figure 1). This is beneficial as glutamate is involved in the catalytic reaction of the key enzyme EctB in the ectoine synthesis pathway, as a co-substrate, and provides an amidogen to L-aspartate-β-semialdehyde (Li et al., 2017).

Since ammonium chloride, as an amino donor, plays an important role in improving ectoine, production, the types, and various concentrations of amino donors were further optimized, as shown in Figures 3C–E. The maximal titer of ectoine (10.2 g/L) was obtained with the addition of ammonium sulfate, which was 1.40- and 1.65-fold higher than that obtained after the addition of ammonium chloride (7.29 g/L) and sodium glutamate (6.18 g/L), respectively. With an increase in the concentration of the amino donor, the ectoine titer showed a trend from increasing to decreasing. It was noted that the maximum titer was obtained when the concentration of NH_4_^+^ (for ammonium salt) or −NH_2_ (for sodium glutamate) was 152 mM, regardless of the presence of ammonium chloride (Figure 3C), ammonium sulfate (Figure 3D), or sodium glutamate (Figure 3E). The concentration of NH_4_^+^ or −NH_2_ had little effect on the growth of bacteria when it was less than or equal to 152 mM. However, when the concentration of NH_4_^+^ or −NH_2_ reached 228 mM, bacterial growth was significantly inhibited, which was viewed as an important factor for the decrease in the total glucose consumption and ectoine production. Although all these three amino donors improved ectoine biosynthesis, ammonium sulfate was the most efficient. This may be attributed to the higher transport efficiency of ammonium sulfate compared with sodium glutamate.

### Effect of NH_4_^+^ on the Relevant Metabolic Pathways

**FIGURE 4:**
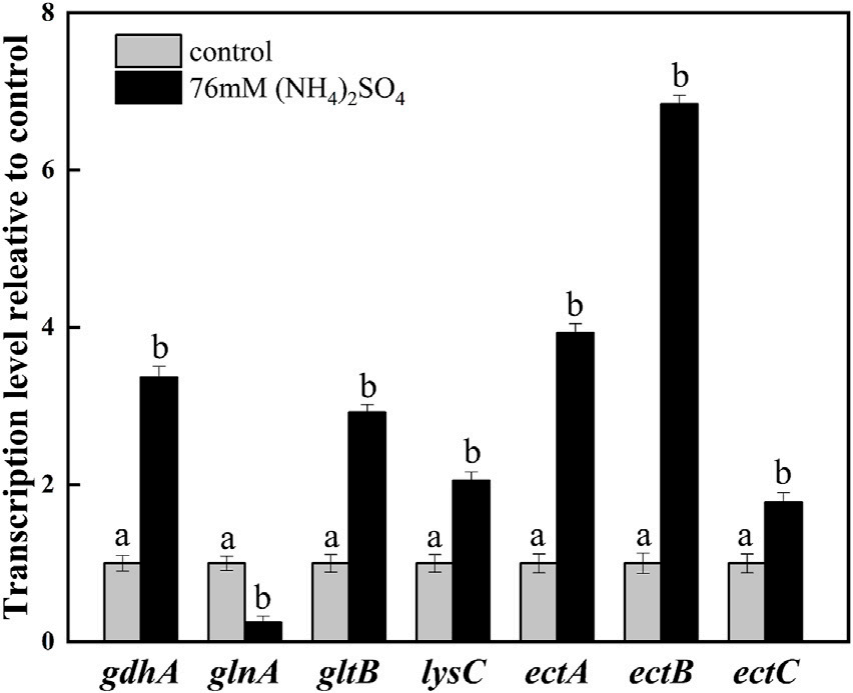
Transcription levels of the key genes in ammonium metabolic pathways and ectoine synthesis. The level of transcription was calculated relative to transcription of the control (0 mM ammonium sulfate), which were defined as 1. Values represent the mean ± SD. Different lowercase letters indicate significant differences at *p* < 0.05.

To exploit the mechanism of NH_4_^+^ on ectoine production, the transcription levels of the related genes were investigated by adding 76 mM ammonium sulfate. The addition increased the transcription levels of *gdhA* and *gltB*. It was observed that the transcription levels of *gdhA* and *gltB* increased by 3.36- and 2.92-fold, respectively, whereas the transcription levels of *glnA* decreased significantly compared with the control (Figure 4). This was expected as it is known that ammonium is mainly assimilated by glutamate dehydrogenase (GDH) encoded by *gdhA*, or glutamine synthetase/glutamate synthase (GS/GOGAT) encoded by *glnA* and *gltB*, respectively (Merrick and Edwards, 1995; Reitzer, 2003). As previously reported, the up-regulation of *gltB* and *gdhA* promoted the synthesis and accumulation of glutamate in the microbial strains during fermentation (Huang et al., 2011). The downregulation of *glnA* on the other hand, indicated the inhibition of glutamine synthesis, but the promotion of glutamate accumulation. Furthermore, glutamate, as an essential amino acid, may be directly involved in the synthesis of ectoine through transamination (Figure 1). Therefore, the addition of ammonium sulfate may enhance the synthesis of ectoine by improving glutamate synthesis.

Aspartokinase (AK), EctA, EctB, and EctC play significant roles in ectoine synthesis. As depicted in Figure 4, the transcription level of the aspartate kinase gene (*lysC*) was upregulated by 2.05-fold, which demonstrated the improved synthesis ability of downstream products in the aspartate metabolic pathway. Significant increases in the transcription levels of *ectABC* which acted as the principal gene cluster in the synthesis of ectoine, were observed. The transcription level of *ectB* was up-regulated by 6.84-fold, which was higher than that of *ectA* (3.93-fold) and *ectC* (1.78-fold). This indicated that EctB was the most important enzyme among the key enzymes involved in the synthesis of ectoine. These results are consistent with that of a previous report (Hillier et al., 2020). It was speculated that the addition of the ammonium sulfate enhanced the supplementation of L-aspartate-β-semialdehyde and glutamate, providing an affluent co-substrate for the key enzyme EctB. At the same time, the strong expression of *ectABC*, especially *ectB*, promoted the flow of substrate in the ectoine synthesis pathway, improving the production of ectoine.

### Optimization of Copy Numbers of Key Genes in Ectoine Synthesis Pathway

**FIGURE 5:**
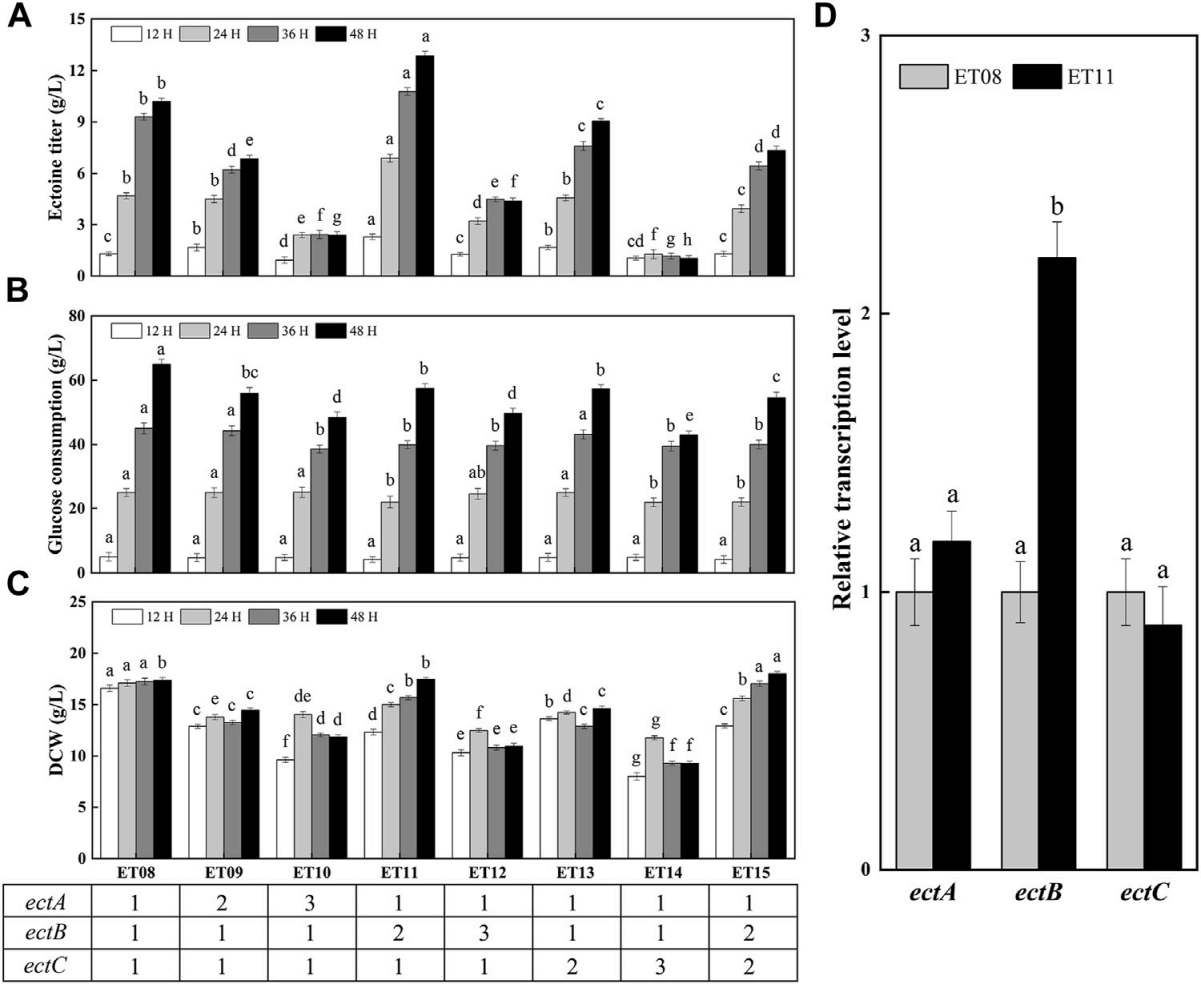
Ectoine production in metabolically engineered *E. coli* with different copy numbers of *ectA*, *ectB*, *ectC*. **(A)** Ectoine production; **(B)** Glucose consumption; **(C)** Cell growth; **(D)** Transcription levels of gene *ectA*, *ectB*, *ectC*. Values represent the mean ± SD. Statistical analysis was performed by Duncan’s test (*p* < 0.05). Different lowercase letters indicate significant differences.

A previous report found that the expression of *ectABC* was not similar among the high-yielding strains, and that the expression ratios of *ectA*, *ectB*, and *ectC* were crucial for obtaining high ectoine production (Gießelmann et al., 2019). In addition, the copy number of the gene may significantly affect the protein expression level. Thus, it is necessary to study the effect of different copy numbers of *ectA*, *ectB*, and *ectC* on ectoine synthesis. As such, the plasmids pTETA, pTETB, pTETC, pTETATA, pTETBTB, and pTETCTC were constructed and introduced into the ET08-N strain, which is the strain ET08 without plasmid, producing ET09-ET14, respectively. The flask fermentation results showed that ectoine titers of ET09 (*ectA*:*ectB*:*ectC* = 2:1:1) and ET10 (*ectA*:*ectB*:*ectC* = 3:1:1) were 6.85 g/L and 2.39 g/L lower than that of ET08 (*ectA*:*ectB*:*ectC* = 1:1:1), respectively (Figure 5A). This indicated that the synthesis of ectoine was not effectively enhanced with an increase of the copy number of *ectA*. It was also noted that as the copy number of *ectA* increased, the DCW was significantly reduced (Figure 5C), revealing that the overexpression of *ectA* had an inhibitory effect on cell growth. Therefore, it was speculated that the overexpression of EctA is toxic to cells.

As the rate-limiting enzyme in the ectoine biosynthetic pathway, the increase in the expression level of *ectB* is of great significance for increasing the production of ectoine (Chen et al., 2015). Gideon Gießelmann et al. (2019) found that all high-yielding strains in the mutant library had high expression levels of EctB. As such, in this study, the copy number of *ectB* was increased 2-fold to generate ET11 (*ectA*:*ectB*:*ectC* = 1:2:1). The production of ectoine by ET11 increased to 12.9 g/L, which was 1.26-fold higher than that of ET08. As shown in Figure 5B, the glucose consumption of ET11 (58 g/L) was lower than that of strain ET08 (65 g/L). As a result, the yield of ectoine based on glucose consumption was increased by 38%. When the copy number of *ectB* was further increased to three copies, the DCW of ET12 (*ectA*:*ectB*:*ectC* = 1:3:1) was significantly decreased to 11 g/L. Glucose consumption and ectoine titers were also reduced. These results indicate that the increase in the copy number of *ectB* can effectively improve ectoine production and enhance the carbon flux to promote ectoine synthesis. A sufficient supply of EctB may form a strong driving force for the subsequent biosynthetic steps. However, a further increase in the copy number did not have a positive effect on the ectoine titer. It is possible that a further increase in the copy number of *ectB* increases the bacterial metabolic burden and causes metabolic imbalance, thereby affecting production and growth.

Similarly, when the copy number of *ectC* was increased, the production of ectoine by ET13 (*ectA*:*ectB*:*ectC* = 1:1:2) and ET14 (*ectA*:*ectB*:*ectC* = 1:1:3) was only 9.05 g/L and 1.04 g/L, respectively. This may be due to the lack of precursors that can be used as a substrate for EctC to be converted into the final ectoine product. Although the total output of ET13 was reduced, the specific production of ectoine by ET13 was 0.62 g/g DCW, which was 1.1-fold higher than that of ET08 (0.59 g/g DCW). Therefore, we further increased the copy number of *ectC* to two copies in ET11 to generate ET15 (*ectA*:*ectB*:*ectC* = 1:2:2). Unfortunately, the specific ectoine production of ET15 was 0.41 g/g DCW, which was significantly reduced compared with that of ET11 (0.74 g/g DCW). In conclusion, ET11 proved to be the most effective at producing ectoine. Transcription analysis showed that the transcription levels of the *ectA*, *ectB*, and *ectC* genes of *E. coli* ET11 were 1.18-, 2.20-, and 0.88-fold that of ET08, respectively (Figure 5D). This indicated that the transcription level of *ectB* in ET11 increased as the copy numbers increased. The optimal copy numbers of *ectA*, *ectB*, and *ectC* were 1, 2, and 1, respectively. The optimal copy number ratio of *ectA*, *ectB*, and *ectC* was found to be 1:2:1. In this case, the transcriptional balancing of *ectA*, *ectB*, and *ectC* might be more suitable for the biosynthesis of ectoine. These results further confirms that the ratio of EctA, EctB, and EctC, and the transcriptional balancing of *ectA*, *ectB*, and *ectC* in the cell, were crucial for the biosynthesis of ectoine, as observed in a previous study using *C. glutamicum* (Gießelmann et al., 2019).

### Fermentation Performance in a 7.5 L Bioreactor

**FIGURE 6:**
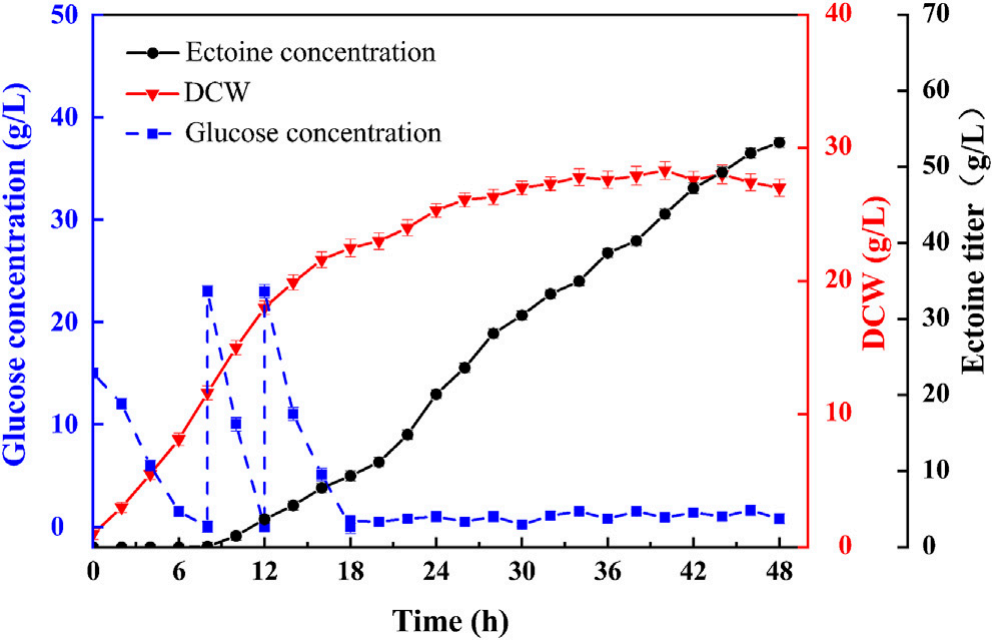
Fed-batch fermentation of ET11 in a 7.5 L bioreactor.

Previously, we reported a fermentation regulation strategy based on the kinetic analysis of ectoine production in fed-batch fermentation (Dong et al., 2021). The two-stage feeding method was combined with a two-step DO control strategy to efficiently increase ectoine production. Based on this fermentation strategy, fed-batch fermentation was implemented in a 7.5 L bioreactor to assess the overall production performance of *E. coli* ET11 with the addition of an exogenous amino donor (Figure 6). Within 18 h of fermentation, the fast-growing strains produced 9.43 g/L of ectoine. When the duration of fermentation was extended to 48 h, 53.2 g/L of ectoine was accumulated with slow bacterial growth. Additionally, the productivity was 1.11 g/L/h. To the best of our knowledge, our work shows the highest ectoine titer and productivity of *E. coli*. Compared with previous studies (Table 2), whole-cell catalysis seems to have certain advantages in yield from substrate, but it requires the addition of extra glycerol and aspartate, which results in high cost of extra substrate and cell culture before the catalytic reaction (Ning et al., 2016). Although *C. glutamicum ectABC* ^*opt*^ (Gießelmann et al., 2019) achieved higher total titer, the yield was only 0.19 g/g, which was lower than our yield (0.33 g/g glucose). Thus, the metabolically engineered *E. coli* strain ET11 and the fermentation strategy of supplementing amino donors display a potential for application in industrial production of ectoine.

**TABLE 2:** Microbial production of ectoine using different fermentative strains or biocatalysts.

Strain	Titer (g/L)	Specific production (g/g DCW)	Yield (g/g)	Productivity (g/L/h)	Process strategy	Reference
*E. coli* Ect05	25.1	0.8	0.11	0.84	Fed-batch	Ning et al. (2016)
*E. coli* BW25113 (pBAD-*ectABC*)	25.1	4.1	-	1.04^c^	Whole-cell catalysis^a^	He et al. (2015)
*E. coli* ECT2	12.7	-	1.27	0.53^c^	Whole-cell catalysis^a^	Chen et al. (2020)
*C. glutamicum ectABC* ^*opt*^	65.3	-	0.19	1.16	Fed-batch	Gießelmann et al. (2019)
*Chromohalobacter salexigens* DSM3043	32.9	0.5	-	1.35	continuous reactors with cell^b^	Fallet et al. (2010)
*E. coli* ET11	53.2	2.0	0.33	1.11	Fed-batch	This work

a Whole-cell catalysis using aspartate and glycerol as substrates at a high cell density.

b A special fermentation process using two continuously operated bioreactors.

c Achieved by calculating reported data.

## Conclusion

In this study, we constructed an efficient strain *E. coli* ET08 by metabolic engineering. By optimizing nutritional element and analyzing the transcription levels, we could conclude that ammonium sulfate, as the optimal amino donor, has a positive effect on ectoine synthesis. Furthermore, optimizing the copy number of *ectA*, *ectB*, and *ectC* was employed to improve ectoine titer to 12.9 g/L in the shake flask. Fed-batch fermentation of *E. coli* ET11 with supplementing amino donor led to an accumulation of 53.2 g/L ectoine. The yield and productivity reached 0.33 g/g glucose and 1.11 g/L/h respectively. It provides a novel strategy for the synthesis of ectoine by engineered strain in industry. This research provides the basis for an effective process for ectoine production and could also be used to produce other high value amino acid derivative.

## Data Availability

The original contributions presented in the study are included in the article/Supplementary Material, further inquiries can be directed to the corresponding authors.
