# Supplementary material for: Metabolic Engineering of Escherichia coli for Ectoine Production With a Fermentation Strategy of Supplementing the Amino Donor
Source: Front Bioeng Biotechnol. 2022 Jan 25;10:824859. doi: 10.3389/fbioe.2022.824859 (PMC8822159; doi:10.3389/fbioe.2022.824859)
Supplement: Supplementary file 1 [file DataSheet1.docx]

Supplementary Material

# Supplementary Tables

**Supplementary Table S1.** Primers used in construction of plasmids and gene knockout.

| **Primer** | **DNA sequence (5’ → 3’）** |
| --- | --- |
| ET01-F | ATGGAATTCGAGCTC**GGTACC**ATGAGCACGCCAATAATACCTTT (***Kpn* I**) |
| ET01-R | CAGGTCGACTCTAGA**GGATCC**TCACCCAGTAGGTGCGGCG (***Bam*H I**) |
| ET02-F | CAGCAAATGGGTCGC**GGATCC**ATGAGCACGCCAATAATACCTTT (***Bam*H I**) |
| ET02-R | GTGGTGGTGGTGGTG**CTCGAG**TCACCCAGTAGGTGCGGCG (***Xho* I**) |
| ET03-F | CAAACGCACCCCGATGATATTGTTTTTACGGCAGATGTTATCGATCAGGCGGTGTAGGCTGGAGCTGCTTC |
| ET03-R | CGGTTCAATTTCCAGTTTCACAGGGTGGCCCAAATGGCGGGCGATTTGCTCACATGGGAATTAGCCATGGT |
| ET04-F | CTGATTAAAAATACCGGAAATCCTCAAGCACCAGGTACGCTCATTGGTGCCGTGTAGGCTGGAGCTGCTTC |
| ET04-R | TTCATCAATATTGCCAACATAGCGCAAAACTTTTCCTTCATCACGGGCCTTACATGGGAATTAGCCATGGT |
| ET06-F | GTGTCCCGTATTATTATGCTGATCCCTACCGGAACCAGCGTCGGTCTGACCGTGTAGGCTGGAGCTGCTTC |
| ET06-R | TTACTGCTGCTGTGCAGACTGAATCGCAGTCAGCGCGATGGTGTAGACGATACATGGGAATTAGCCATGGT |
| ET07-H1-F | TTCATCTAATGCAATACGTGTCCCG |
| ET07-H1-R | GAAGCAGCTCCAGCCTACACTTGCAGGTACTTCTTGTCGTACTGT |
| ET07-H2-F | ACCATGGCTAATTCCCATGTAACTTAAGCAATCTGGAAAAAGGCG |
| ET07-H2-R | CAGTTCACCATTAGACAGTTTGCCT |
| ET08-H1-F | TTGCGTAACCTTTTCCCTGGAACGT |
| ET08-H1-R | GAAGCAGCTCCAGCCTACACTTCAGATTCGGTTTTCGGTCCGATG |
| ET08-H2-F | ACCATGGCTAATTCCCATGTTGCACTGGTACCGAGCGGCACTACT |
| ET08-H2-R | ACCCAGGATTACCGCGCCCAGTACC |
| pKD3-up | GTGTAGGCTGGAGCTGCTTC |
| pKD3-down | ACATGGGAATTAGCCATGGT |
| ET03-out-F | TTCAGCACCGATACCGATCTCACCG |
| ET03-out-R | TCATGCAACCAGCGACTAACCGCAG |
| ET04-out-F | CCTGACAGTGCGGGCTTTTTTTTTC |
| ET04-out-R | ACCCGACGCTCATATTGGCACTGGA |
| ET06-out-F | CGGCGGTAACGAAAGAGGATAAACC |
| ET06-out-R | CGCAAAGCTGCGGATGATGACGAGA |
| ET07-out-F | ATGAAACTCGCCGTTTATAGCACAA |
| ET07-out-R | TTAAACCAGTTCGTTCGGGCAGGTT |
| ET08-out-F | TCTCAACTTAAAGACTAAGACTGTC |
| ET08-out-R | TACAAATTAATTCACAAAAGCAATA |
| TETA-*trc*-F | CGCACCTACTGGGTGAGGATCCTTGACAATTAATCATCCGGCTCGTATAATGT |
| TETA- *trc*-R | GGTAAAAGGTATTATTGGCGTGCTCATAGATCTGGTCTGTTTCCTGTGT |
| TETA-*ectA*-F | ACACAGGAAACAGACCAGATCTATGAGCACGCCAATAATACCTTTTACC |
| TETA- *ectA* -R | GCTTGCATGCCTGCAGGTCGACTTACATGCTGTCTGTTTGAAATGGACC |
| TETATA-F | AACAGACAGCATGTAAGTCGACTTGACAATTAATCATCCGGCTCGTATAATGT |
| TETATA-R | AAACAGCCAAGCTTGCATGCCTGCAGGTTACATGCTGTCTGTTTGAAATGGACCTATACG |
| TETB-*trc*-F | CGCACCTACTGGGTGAGGATCCTTGACAATTAATCATCCGGCTCGTATAATGT |
| TETB- *trc*-R | GTTCAAGCGTTTGGGTCTGCATAGATCTGGTCTGTTTCCTGTGT |
| TETB-*ectB*-F | ACACAGGAAACAGACCAGATCTATGCAGACCCAAACGCTTGAAC |
| TETB- *ectB* -R | GCTTGCATGCCTGCAGGTCGACTCACGCTTGGATAACAGCATTGACAG |
| TETBTB-F | TGTTATCCAAGCGTGAGTCGACTTGACAATTAATCATCCGGCTCGTATAATGT |
| TETBTB-R | AAACAGCCAAGCTTGCATGCCTGCAGGTCACGCTTGGATAACAGCATTGACAG |
| TETC-*trc*-F | CGCACCTACTGGGTGAGGATCCTTGACAATTAATCATCCGGCTCGTATAATGT |
| TETC- *trc*-R | GTGCTTCTTCAAGGTTACGAACGATCATAGATCTGGTCTGTTTCCTGTGT |
| TETC-*ectC*-F | ACACAGGAAACAGACCAGATCTATGATCGTTCGTAACCTTGAAGAAGCAC |
| TETC- *ectC* -R | GCTTGCATGCCTGCAGGTCGACTCACCCAGTAGGTGCGGC |
| TETCTC-F | CGCACCTACTGGGTGAGTCGACTTGACAATTAATCATCCGGCTCGTATAATGT |
| TETCTC-R | AAACAGCCAAGCTTGCATGCCTGCAGGTCACCCAGTAGGTGCGGCG |
| TETBTC-F | TGTTATCCAAGCGTGAGTCGACTTGACAATTAATCATCCGGCTCGTATAATGT |
| TETBTC-R | AAACAGCCAAGCTTGCATGCCTGCAGGTCACCCAGTAGGTGCGGCG |

**Supplementary Table S2.** Primers used in RT‑qPCR.

| **Primer** | **DNA sequence (5’ → 3’）** |
| --- | --- |
| *gdhA* (RT)-F | CCTTTCATTTGGCGGCAGTC |
| *gdhA* (RT) -R | TTTCGATAGCGTACTGGGCG |
| *gltB* (RT)-F | TCTCCGTGAAGCTGGTTTCC |
| *gltB* (RT)-R | CTGCTGGGTTTCAACAAGCC |
| *glnA* (RT)-F | CGGAGAAGAAACCACCGGAA |
| *glnA* (RT)-R | TTGGCGGCGTAATCAAACAC |
| *lysC* (RT)-F | GCCAGACCTTCTTCCACCTC |
| *lysC* (RT)-R | GAAGTTTTCGGCATCCTCGC |
| *ectA* (RT)-F | CAGTCGCTGATGCTGTGGTT |
| *ectA* (RT)-R | AACTGGGTTGCCAGCAGTAG |
| *ectB* (RT)-F | CGCACGGGCAAGTTCTTTAG |
| *ectB* (RT)-R | CATCGCCAAGTTGAAGCCAC |
| *ectC* (RT)-F | ACCCGTATTTTCCCAGGCAC |
| *ectC* (RT)-R | CAGGTGTTCGTCGTGCTGAT |
| 16s(RT)-F | CTGGAACTGAGACACGGTCC |
| 16S(RT)-R | GGTGCTTCTTCTGCGGGTAA |

# Supplementary Figures


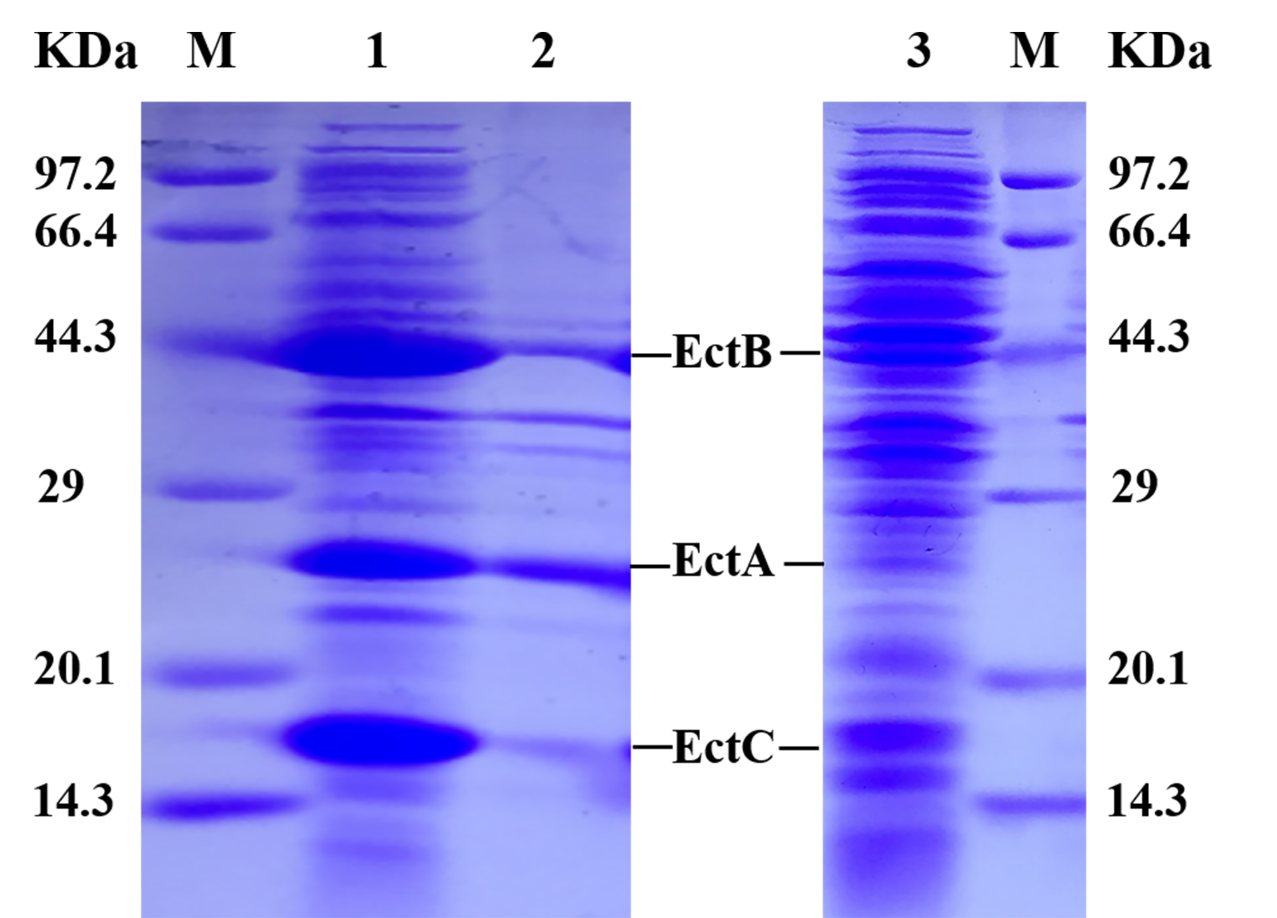


**Supplementary Figure S1.** The expression of EctA, EctB, and EctC in ET00, ET01, and ET02. M: molecular weight markers; lane1: ET02; lane2: ET01; lane3: ET00.

**

**

**Supplementary Figure S2.** Comparison of organic acid in recombinant *E. coli* strains. Values represent the mean ± SD.
